# Implementation of shared decision-making in oncology: development and pilot study of a nurse-led decision-coaching programme for women with ductal carcinoma in situ

**DOI:** 10.1186/s12911-017-0548-8

**Published:** 2017-12-06

**Authors:** Birte Berger-Höger, Katrin Liethmann, Ingrid Mühlhauser, Anke Steckelberg

**Affiliations:** 1 MIN-Faculty, Unit of Health Sciences and Education, University of Hamburg, Martin-Luther-King-Platz 6, D-20146 Hamburg, Germany; 2 Department of Pediatrics and Institute of Medical Psychology and Sociology, University Medical Center Schleswig-Holstein, Schwanenweg 20, D-24105 Kiel, Germany; 3 Institute for Health and Nursing Science, Martin-Luther-University Halle-Wittenberg, Magdeburger Str. 8, D-06112 Halle (Saale), Germany

**Keywords:** Decision making, Decision support techniques, Patient participation, Breast neoplasms, Carcinoma, intraductal, non-infiltrating, Evidence-based medicine, Oncology nursing, Interprofessional relations, Professional-patient relations, Feasibility studies

## Abstract

**Background:**

To implement informed shared decision-making (ISDM) in breast care centres, we developed and piloted an inter-professional complex intervention.

**Methods:**

We developed an intervention consisting of three components: an evidence-based patient decision aid (DA) for women with ductal carcinoma in situ, a decision-coaching led by specialised nurses (breast care nurses and oncology nurses) and structured physician encounters.

In order to enable professionals to gain ISDM competencies, we developed and tested a curriculum-based training programme for specialised nurses and a workshop for physicians. After successful testing of the components, we conducted a pilot study to test the feasibility of the entire revised intervention in two breast care centres. Here the acceptance of the intervention by women and professionals, the applicability to the breast care centres’ procedures, women’s knowledge, patient involvement in treatment decision-making assessed with the MAPPIN’SDM-observer instrument MAPPIN’O_dyad,_ and barriers to and facilitators of the implementation were taken into consideration. We used questionnaires, structured verbal and written feedback and video recordings. Qualitative data were analysed descriptively, and mean values and ranges of quantitative data were calculated.

**Results:**

To test the DA, focus groups and individual interviews were conducted with 27 women. Six expert reviews were obtained. The components of the nurse training were tested with 18 specialised nurses and 19 health science students. The development and piloting of the components were successful. The pilot test of the entire intervention included seven patients. In general, the intervention is applicable. Patients attained adequate knowledge (range of correct answers: 9–11 of 11). On average, a basic level of patient involvement in treatment decision-making was observed for nurses and patient–nurse dyads (M(MAPPIN-O_dyad_): 2.15 and M(MAPPIN-O_nurse_): 1.90). Relevant barriers were identified; physicians barely tolerated women’s preferences that were not in line with the medical recommendation. Classifying women as inappropriate for ISDM due to age or education led physicians to neglect eligible women during the recruitment phase.

**Conclusion:**

Decision-coaching is feasible. Nevertheless, there are some indications that structural changes are needed for long-term implementation. We are currently evaluating the intervention in a cluster randomised controlled trial in 16 breast care centres.

**Electronic supplementary material:**

The online version of this article (10.1186/s12911-017-0548-8) contains supplementary material, which is available to authorized users.

## Background

Women with breast cancer want to participate in treatment decision-making [1]. Treatment options, particularly in oncology, may differ in their risk-benefit profiles. Adequate and individualised counselling by members of the healthcare team is needed in order to arrive at the best decision together with the patient. Ideally, women are enabled to make informed treatment decisions based on evidence-based information and according to their individual preferences and values [2, 3]. This aim could be reached by providing evidence-based patient information combined with informed shared decision-making (ISDM). Evidence-based patient information (EBPI) provides information about the disease, the treatment options and its potential benefits and harms (if possible displayed in absolute risk rates and absolute risk reductions) to enable patients making informed decisions. All information is based on best available evidence [4, 5]. If an EBPI is supplemented with a value clarification tool, it is often called a patient decision aid (DA) [6].

In Germany, ISDM [7, 8] has a legal and ethical basis and is explicitly included as an objective in medical guidelines [9–13]. However, ISDM is still not universally implemented, and health-related decisions are often made exclusively by healthcare teams [1, 14]. The implementation of ISDM is hampered by barriers such as time constraints and the prevailing traditional paternalistic patient-physician relationship [15]. Furthermore, evidence-based decision aids (DA) that provide risk information on the benefits and harms of cancer treatments according to the criteria for evidence-based patient information (EBPI) [4] are lacking for most decisions in oncology [6].

Currently, most types of ISDM-training address physicians only [16]. Decision-coaching by nurses is an alternative approach to implementing ISDM [17]. Before women make their final decisions together with the physician, the nurse supports the decision-making process by providing and discussing a decision aid with the women concerned [18, 19].

In Germany, breast care nurses’ (BCN) main tasks comprise educating and counselling of women for supportive care and treatment, as well as the coordination of the care process [20, 21]. They are not yet involved in treatment decision-making with patients.

Our aim was to develop and pilot a new approach: an inter-professional ISDM programme for specialised nurses (BCN and oncology nurses) and physicians to enable them to provide ISDM in breast care centres.

With this programme, basic competencies of decision-coaching combined with DA are imparted to nurses. We used ductal carcinoma in situ (DCIS) as an example. DCIS is a cell abnormality restricted to the milk ducts. The associated risk of invasive cancer is difficult to quantify and probably depends on histological characteristics such as grading and comedo necrosis. The natural course of the disease is unknown. Medical guidelines recommend either breast-conserving surgery with radiation or mastectomy [12]. In Germany, the diagnosis of DCIS has increased since the national mammography programme started in 2005 [22]. About 20% of the findings in mammography screening are DCIS [22]. The increasing prevalence of DCIS is associated with over-diagnosis and over-treatment [23–25].

## Methods

**Fig. 1:**
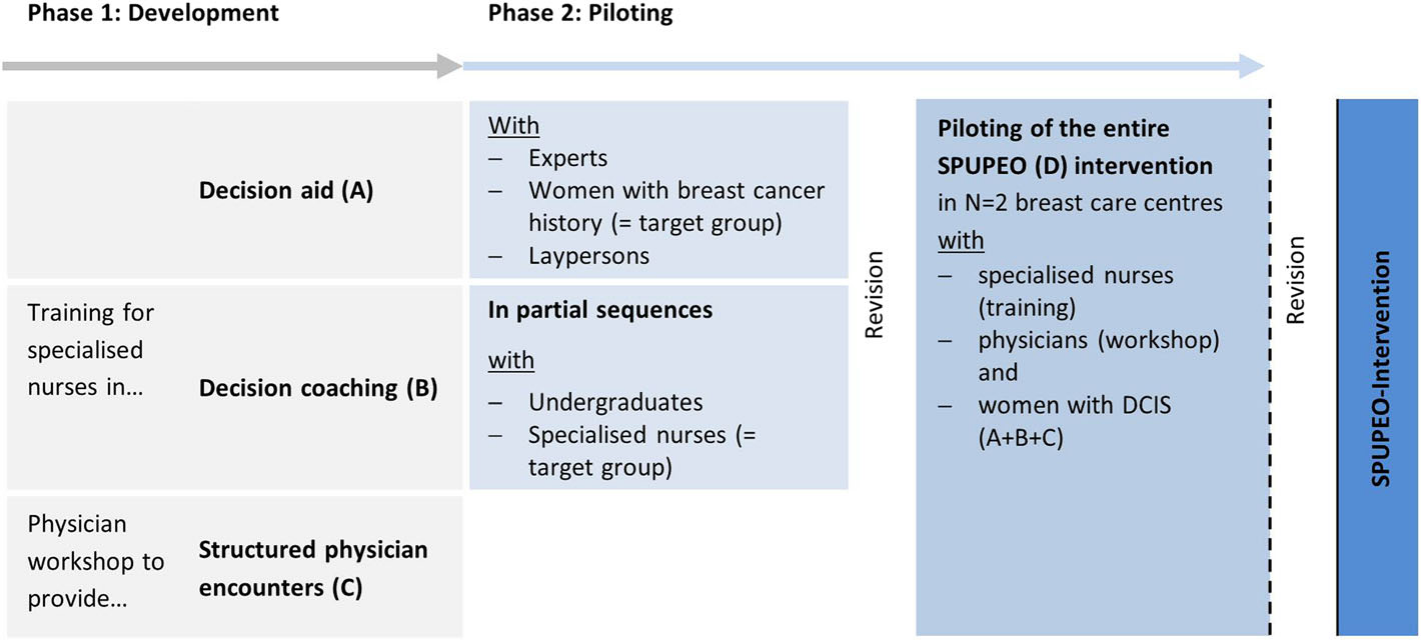
The SPUPEO-intervention: Development and piloting of single components and entire intervention

We developed and pilot tested a complex intervention in accordance with the UK Medical Research Council’s guidance (phase 1 and 2) [26]. Our results are reported in line with the revised criteria for Reporting the Development and Evaluation of Complex Interventions in healthcare (CReDECI 2) [27] (see Additional file 1). Under the title ‘Specialised nurses to support informed shared decision-making in oncology’ (the acronym SPUPEO refers to the German translation), the intervention comprised A) an evidence-based DA for women with DCIS, B) nurse-led decision-coaching and C) structured physician encounters (see Fig. 1). The programme of the present study constitutes a prototype that could easily be supplemented with further modules related to other treatment decisions for breast cancer. In addition, it constitutes a prototype that could also be transferred to other oncological settings.

Most physicians and nurses lack competence in ISDM. Therefore, we prepared single components in a two-phase study comprising an evidence-based DA, a training for nurses, and a physician workshop (see Fig. 1) in preparation for a pilot study testing the entire intervention with patients.

All the components were developed taking the theory of planned behaviour [28, 29], the theory of cognitive dissonance [30], and further experiences reported in the literature [31] into consideration. According to the theory of planned behaviour, a woman facing a treatment decision on DCIS could be influenced by her own attitudes, by the preferences of related parties (subjective norms) and her perceived ability to manage the decision. The same applies to healthcare team members concerning the adoption of ISDM behaviour. In the following section, we report the development and pilot testing of the three components (A, B, C) and the piloting of the entire intervention with women with DCIS (D).

## A) Evidence-based decision aid for women with DCIS

### Phase I: Development of the decision aid

The project started in 2012. We conducted a systematic review of existing DAs for women with breast cancer (see Additional file 2, section I) and critically appraised the 27 identified DAs using the International Patient Decision Aids (IPDAS)-instrument [32] and the EBPI-criteria [4]. Two of the identified DAs addressed the treatment decision for DCIS [33–35]. None included the option “watchful waiting” or had been evaluated in a randomised controlled trial (RCT). Therefore, we decided to develop a DA following the EBPI-criteria [4]. We could not assess the evidence underlying the decision aids since there were no reports available about the development process or research.

In addition, two patient representatives and two experts (one nursing scientist who was involved in the training of breast care nurses and one experienced breast care nurse who was involved in the treatment and care process of women with DCIS) were concerned in the selection of topics for our first DA for women with breast cancer. Due to the introduction of the national mammography screening programme and concomitant over-diagnosis, treatment decisions related to DCIS were considered highly relevant. The format is a brochure containing medical information in text, tables and pictograms and a value clarification tool. For more information on the development process, see Additional file 2.

### Phase II: Pilot testing

#### Methods

To test the comprehension and acceptability of the DA, we conducted four focus groups with women with no history of breast cancer (*n* = 19 in groups of 3–6), moderated by at least two researchers; three individual interviews with women with no history of breast cancer, conducted by one researcher; and one separate focus group with women with a history of breast cancer (*n* = 4). The women were recruited through advertisements in newspapers, online portals and via self-help groups.

The women were aged between 21 and 74 years. Although we tried to sample according to educational background, most of the participants had a higher education (lower secondary school *n* = 2, secondary school *n* = 6, upper secondary school *n* = 9, college/university diploma *n* = 9). The focus groups lasted between 90 and 120 min.

Interviews were transcribed verbatim and field notes were taken by the interviewers. Qualitative content analysis was then conducted according to the approach of Mayring [36]. One researcher coded the transcripts using MAXQDA® 10 [37] and the results were discussed by two researchers. The results guided the revision in an iterative process.

We also obtained six reviews of a pre-final version of the DA from a patient representative, a nurse scientist, a journalist, a gynaecologist and two experts on patient information, evidence-based medicine and oncology guidelines.

#### Results

The women understood the objectives and the content of the DA. The layout was well accepted and most participants appreciated the female writing-style. Women rated the value clarification tool as helpful. Nevertheless, both the women and the experts criticised some aspects (see Table 1). The final version of the DA contained 64 pages. We developed an additional decision guidance workbook for patients, which included the value clarification tool, to structure the decision process during the decision-coaching sessions and to document individual diagnoses, personal preferences and notes (see Additional file 2, section IV).

**Table 1:** Results of focus groups, expert reviews and revision process

Commentary of focus group participants (FG) and expert reviews (ER)	Revision
Length/completeness	
**FG:** Overwhelming amount of information	Text reduction; additional information is given as online resource linked with QR-codes for interested readers
**FG:** Request for information about lifestyle interventions to influence the course of the disease	Chapter with the requested information was added
**ER:** Information about overdiagnosis and overtreatment might be an information overload	Information was retained since it is relevant for treatment decisions
**ER:** Inclusion of the Van-Nuys-Prognostic Index [67] was recommended	Suggestion was rejected: Index has not been validated prospectively by now [68]; applies only to recurrence risk after treatment. Instead, we listed factors associated with a higher recurrence risk (grading, size of lesion etc.).
Information quality	
**ER:** Radiation procedures changed over time in dose and application so that the external validity of study results could be low	Common problem in oncology research; the best available evidence is presented; limitation of generation
**ER:** Data on re-operation rates after mastectomy were quite heterogeneous. Experts recommended removing these data	Data have been removed and a hint was replaced that valid data are not available
Comprehensibility	
**FG:** Misunderstandings of figures and text passages	Figures have been rearranged and the text passages were revised
**FG:** Challenging information: dissent between positive results and poor study validity	Contradiction could not be solved: If required, the dissent can be explained by the decision coaches during the coaching
**FG:** Redundant information in text and tables was annoying for women with higher education	Some women favoured the written presentation of risks, some preferred the tables. Therefore, redundancy was kept.
Acceptability	
**FG:** Breast cancer survivors valued the photos after breast cancer surgery as realistic but not aesthetic	Photos originated from the documentation of the treatment process in medical records; photos are available on demand as additional online resource
**ER**: Option watchful waiting polarizes: - Patient representatives pointed out its importance - Ebm-experts disbelieved that this could be a real option due to the missing evidence and associated uncertainties	The option was retained
**ER:** The term ductal ‘carcinoma’ in situ may cause anxiety	We replaced ‘*ductal carcinoma’* by the abbreviation DCIS. DCIS was not classified as a pre-stage of invasive breast cancer due to the unknown natural course [69, 70]

## B) Decision-coaching led by nurses

### Phase I: Development of the nurse training curriculum

To enable the nurses to provide decision-coaching with suitable material, we developed and piloted a curriculum based on our research group’s previous work on curricula for evidence-based medicine and ISDM for patient representatives and non-medical health professionals [38–40]. The curriculum was developed according to the six-step approach for medical education curriculum development proposed by Kern et al. [41]. We also considered the curriculum for a post-registration course in cancer nursing developed by the European Oncology Nursing Society (EONS) [21].The nurse training comprised two modules with 2 days of practical exercises in between. Module I (2 days) imparts competencies on basics of medical decision-making and judging the quality of information. In module II (1–2 days according to group sizes), the participants acquire competencies in ISDM and decision-coaching using DA. We used specific breast cancer topics as examples for the training modules. For a detailed overview of the learning goals, content and educational strategies, see Additional file 3.

### Phase II: Pilot testing of single components and of the entire training

#### Methods

The feasibility and acceptability of the single components were tested from March to August 2013. Module I was divided into two parts. The first part contains mainly basic information on evidence-based medicine and evidence-based nursing. The second part comprises critical appraisal of patient information material and risk communication. We used training sessions for reciprocal observation of the instructors by taking field notes in lectures and documenting the participants’ learning results (e.g. worksheets) in order to assess the appropriateness of the time schedule, learning methods and comprehensibility. At the end of each module, we conducted focus groups to get feedback from the participants with the attention on comprehensibility and appropriateness of the learning methods. At the end of the second module, we conducted focus groups to elicit the nurses’ thoughts and assumptions about the feasibility of implementing the intervention in their daily practice.

To describe the sample, we collected socio-demographic data and information on their work experience. The interviews were audiotaped and transcribed verbatim using the F4© software [42].

We recruited three samples (BCNs, oncology nurses and health science students) that could easily be accessed and were comparable with the target group.

For the first part of module I, health science and education students in their second semester (*N* = 19) were recruited from the University of Hamburg. They were aged between 21 and 33 and had completed vocational healthcare training. Four had a nursing background and five had experience in breast care. For the second part of module I, oncology nurses (*N* = 12) were recruited from a training institute that provides advanced training for nurses. They were aged between 25 and 52. Most of them had more than 10 years of experience in nursing and had worked in various disciplines, including one in a breast care centre. For module II, BCNs (*N* = 6) were recruited using a mailing list from a breast care nurse network. They were aged between 36 and 62. All of them had more than 16 years of experience in nursing; half of them were employed as BCNs and were excused from their work on the ward.

Participation in the nurse training was free of charge, and the participants did not receive incentives or allowances. The components were optimised through an iterative process.

We conducted a qualitative content analysis [43] using the MAXQDA 10© software [37] to identify categories of feasibility and acceptance [43]. Categories were derived by one researcher from the transcripts of the focus groups, the protocols of the reciprocal observation and the documented learning results. The results were discussed by two researchers. The data were combined by between-methods triangulation [44]. To reduce complexity, only the main feasibility and acceptability results are reported. The complete coding scheme is available on request.

#### Results

The group size, time schedule and selection of methods were adequate and well accepted by the nurses. The duration of the training sessions varied according to group size, their working experience in the field of breast cancer and whether nurses had an advanced training as oncology or as breast care nurses.

We also identified important challenges and barriers to implementing the ISDM coaching by nurses.

The treatment recommendations are usually made by a multi-professional healthcare team during tumour board meetings whereby individual patient preferences are rarely considered [45]. The nurses were not aware that the regularly held multidisciplinary tumour boards give recommendations rather than actual prescriptions. They regarded the tumour board recommendation as a final decision that was not negotiable. Some nurses did not ascribe priority to the assessment of women’s preferences, but felt obliged to try to convince women to follow medical recommendations. Further possible barriers were of a structural nature or specific to the economic situation of a particular hospital. For example, in some hospitals the marketing department stipulates the release of specific patient information material.

A quiet room and sufficient time were considered indispensable for decision-coaching. Finally, the nurses asked for structured guidance for the decision-coaching sessions.

#### Revision

The training was revised according to our findings. We scheduled extra time for discussion and reflection on the nurses’ experiences and how their own values and attitudes influenced women’s decision-making. The originally generic decision guidance was adapted to the specific requirements of DCIS. We added a section in which diagnostic results relevant to DCIS treatment decision-making can be documented. In addition, a list of possible decision-leading criteria was supplemented (e.g. preservation of the breast, short treatment duration or low risk of invasive cancer and recurrence). Prompt cards for the nurses were developed to provide verbal prompts for decision-coaching.

### Pilot testing of the entire nurse training

#### Methods

We piloted the entire intervention in two certified breast care centres with specialised nurses, physicians and women with DCIS. In preparation of the entire testing, we pilot-tested the entire nurse training with the specialised nurses at the participating centres. The recruitment process, the eligibility criteria and the sample are described in detail in section D.

The nurse training was conducted between November 2014 and January 2015. The first module was delivered to all of the nurses; the second module was split into two groups. The time between the modules was three and 9 weeks. We focused on the feasibility and acceptance of the training, the appropriateness of the time schedule, the content and the anticipated barriers to implementation. In addition to the previously applied data collection methods, the nurses were asked to give a structured written feedback about their attitude towards ISDM and their expectations for the training at baseline (31 items). After both training modules, written and verbal feedback of their attitudes to ISDM and their satisfaction with the training was obtained (45 items). The items were constructed mainly as a visual analogue scale and open questions. In addition, we assessed their knowledge by using questionnaires after module I, and before and after module II. These questionnaires comprised 72 items, including a shortened version of a patient’s risk knowledge test that was developed to assess patients’ knowledge after the intervention. The knowledge items were constructed mainly in a multiple-choice format. The feedback forms were analysed descriptively by two researchers (BBH, KL). Due to the small sample size, we calculated only the range of correct answers for the knowledge test.

#### Results

Overall, the nurses were interested in and positive about their new roles as decision coaches. They appreciated that the training helped them to understand research studies and risk information. However, some nurses were more reluctant and expressed a lack of confidence in their qualifications to provide decision-coaching. Indeed, the physicians appeared to have more confidence in the nurses. After the training, the nurses had revised some of their beliefs. They no longer considered it necessary to persuade women to follow the tumour board recommendations. Practical exercises and role-playing within the training sessions were associated with the nurses’ enhanced self-confidence.

The nurses had adequate knowledge, with scores ranging from 51 to 62 out of 72. In particular, disease and risk knowledge about DCIS were high, with scores ranging from 9 to 11 out of 11.

Overall, the nurses judged the teaching modules, time schedule and comprehensibility to be appropriate, and they were satisfied with the training. The opportunity to exchange experiences and the small size of the group were particularly welcome aspects. During the training, it became apparent that additional material was needed to present the essential information about treatment options in a structured manner. The nurses highly appreciated the decision guidance and the DA, but they considered time constraints and work overload as relevant barriers to the implementation of the coaching model in routine practice.

#### Revision

No major revisions were necessary. A minor revision was made to two worksheets, which were shortened. Prompt cards for each decision option were supplemented by fact sheets, which were based on the DA and which summarised the main risk information.

## C) Structured physician encounters

### Phase I: Development of a physician workshop

In order to prepare physicians for providing structured encounters after the nurse coaching, we developed and piloted a physician workshop. The aim was to ensure that the whole team was committed to the idea of inter-professional ISDM. The workshops also provided the physicians with insights into the DA provided to the women with DCIS. The workshop consisted of lectures and discussions. According to the theoretical background, we emphasised physicians’ concerns about ISDM and the provision of risk information. We addressed known barriers to the implementation of ISDM in general, and asked the physicians to disclose their personal concerns. The workshops were scheduled for 2 h and were performed in the physicians’ individual workplaces to ensure the full participation of the respective healthcare teams. The specific goals of the physician workshops are outlined in Additional file 4.

### Phase II: Pilot testing of the physician workshop

#### Methods

We piloted the physician workshop (BBH) in two certified breast care centres (for a detailed description see section D), focusing on the appropriateness of the time schedule, the content, the acceptability of the intervention and the anticipated barriers. Physicians were asked for written and verbal feedback about their attitudes towards ISDM and their satisfaction with the workshop, the DA, the decision guidance and fact sheets, as well as the anticipated barriers to and facilitators of implementation. As a result, we developed structured feedback forms that were completed by the physicians at three time points: prior to the workshop (19 items), after the workshop (5 items) and after decision-coaching of women with DCIS (11 items). The physician workshops were assessed by an expert (AS) with regard to content and feasibility. The feedback forms and observational data were analysed descriptively by two researchers (BBH, KL).

#### Results

The information content, comprehensibility, time requirement and patient information material were regarded as helpful, accurate and adequate. The physicians appreciated being given sufficient time for critical discussion. However, they opted for more in-depth discussion of the treatment options and the related information provided to patients. They raised concerns about offering women options that were not in line with the German medical guidelines. The physicians were also worried that women’s preferences might not match their own recommendations. They did not consider ‘breast conserving surgery without radiation’ an option, and they were concerned that women might falsely assume that by avoiding radiation they could reduce adverse treatment effects. In contrast, they anticipated that women would not opt for ‘watchful waiting’, because laypersons consider doing nothing as inferior to any medical intervention.

The physicians recommended removing information about HER2- and hormone-receptor status from the decision guidance because these diagnostic parameters are not relevant for treatment decisions. They were concerned that women might get the impression that physicians withhold treatment. The physicians emphasised that women attribute high importance to the physician’s treatment recommendation. The time and personal expenditure for decision-coaching was perceived as an expected barrier for the implementation.

#### Revision

We revised the decision guidance according to the suggestions. Future workshops will include extra time for discussing the treatment options and barriers.

## D) Testing the entire intervention with women with ductal carcinoma in situ

In the final step, we piloted the entire intervention with patients in two certified breast care centres [46, 47]. Our aim was to test the feasibility of the intervention and to identify the barriers and facilitators under routine conditions in preparation for an RCT. We tested the feasibility of the recruitment strategy for women with DCIS. We focused on the level of patient involvement that the nurses achieved in the decision-coaching, the women’s acceptance of the intervention, its applicability in the procedures in breast care centres and the usefulness of the information material for decision-coaching.

### Methods

#### Recruitment

A recruitment letter was sent out to 12 certified breast care centres in Berlin, Germany. Breast care centres were eligible if they employed at least one BCN or oncology nurse as required by certification guidelines. Nurses were eligible if they had advanced training as a BCN or oncology nurse. Physicians were eligible if they were involved in providing information to women facing a primary treatment decision for DCIS.

The attending physicians in each breast care centre recruited up to six women with DCIS. Women with DCIS were eligible if they were at least 18 years old and faced a treatment decision concerning a primary, histologically confirmed DCIS. The women needed sufficient German language skills because all of the information was provided in German. Women were excluded if they were pregnant, had a known BRCA 1/2 mutation or if they had a previous diagnosis of breast cancer, lobular carcinoma in situ or DCIS. Women with contraindications for radiation were not included.

Written informed consent was obtained from every participant.

#### Procedures

In an initial consultation, the physicians disclosed the diagnosis. The physicians were not supposed to give a treatment recommendation during this consultation. Afterwards, the women were given the DA by the specialised nurse with a new appointment for at least one (max. two) decision-coaching session. During the nurse-led decision-coaching, the treatment options were discussed taking the women’s preferences into consideration. After sufficient time for consideration (as determined by the women), an appointment with a physician was scheduled to make final treatment decisions and follow up arrangements.

#### Data collection

The baseline characteristics of women and diagnostic parameters (grading etc.) were assessed using a questionnaire.

Professionals’ expectations and worries before the intervention: Physicians’ and nurses’ expectations prior to the intervention were documented by an observer (AS) during the physician workshop and nurse training.

Recruitment strategy for women with ductal carcinoma in situ: In order to test the feasibility of the recruitment strategy, the physicians had to fill in a recruitment sheet in which the eligibility criteria for women were assessed.

Women’s expectations and acceptance of decision-coaching: The women were asked for a structured written feedback about their expectations and acceptance of decision-coaching before (21 items) and after the coaching session and the final physician consultation (25 items).

Women’s knowledge: Prior to the intervention women were asked to estimate their knowledge about breast cancer using the structured feedback form. After the coaching session, the women were given a 15-item, multiple-choice knowledge test.

Patient involvement in treatment decision-making during decision-coaching: The decision-coaching sessions were videotaped in order to assess patient involvement in treatment decision-making. The camera focused on the nurse and the materials used, but not on the women with DCIS.

Use of the DA, patient guidance and fact sheets: The video recordings were analysed for the use of materials (DA, decision guidance, fact sheets). In addition, a copy of the decision guidance was made and feedback from women, nurses and physicians was obtained.

Applicability of the procedures in breast care centres: To assess the applicability of the procedures in breast care centres, the time needed for decision-coaching and the intervals between consultations and decision-coaching were documented. In addition, written feedback was obtained from nurses and physicians at the end of the study.

Professionals’ attitude toward the intervention: At the end of the study, structured written feedback was obtained from the nurses and physicians about their inter-professional collaboration and their attitudes toward the intervention.

Barriers and facilitators of the implementation: During the recruitment phase telephone interviews with the nurses were conducted and field notes taken to identify facilitators and barriers. At the end of the study, structured written feedback was obtained from the nurses and physicians about the implementation barriers and facilitators.

The items of the structured feedback forms were mainly constructed as visual analogue scales and open-ended questions.

#### Data analysis

The observer-based instrument of the MAPPIN’SDM-inventory (Multifocal Approach to the ‘Sharing’ in SDM) [48] was applied to measure the extent of patient participation in the decision-coaching sessions (possible range: 0 ‘*competence was not observed*’ to 4 ‘*excellent performance’*). The inventory comprises a set of nine indicators: six indicators outline the chronological order of an SDM talk and three contain meta-communicative components (see Table 2). Indicators 1 to 4 are a mandatory part of the decision-coaching and indicators 5 to 6 are usually scheduled as part of the final physician consultation, where there may be some overlap. Six trained observers (five female, one male) independently rated the ISDM behaviour of the nurse and the patient as well as the interaction of the dyad (nurse and patient), and came to a consensus. Finally, we calculated the mean values of each indicator per unit (nurse, patient and dyad) (M_indicator1_(MAPPIN-O_nurse_), … M_indicator9_(MAPPIN-O_nurse_), analogue for MAPPIN-O_patient_ and MAPPIN-O_dyad_) and the mean total value of all indicators per unit (M(MAPPIN-O_nurse_), M(MAPPIN-O_patient_), M(MAPPIN-O_dyad_). We calculated the range of correct answers in the knowledge test.

**Table 2:** MAPPIN’SDM-observer instrument [48]

			Measurement unit		
MAPPIN’SDM-Indicator		Description	Nurse/Physician (focuses the professional behaviour)	Patient (focuses the patient behaviour)	Dyad (takes both patient and professional behaviour into account)
Defining problem		To draw attention to an identified problem as one that requires a decision-making process	MAPPIN-O_nurse1_	MAPPIN-O_patient1_	MAPPIN-O_dyad1_
SDM key message		To state that there is more than one way to deal with the identified problem	MAPPIN-O_nurse2_	MAPPIN-O_patient2_	MAPPIN-O_dyad2_
Discussing the options	a) structure	To structure the discussion of the options in a way that is easy to understand and easy to remember.	MAPPIN-O_nurse3a_	MAPPIN-O_patient3a_	MAPPIN-O_dyad3a_
	b) content	To explain and discuss the pros and cons of the different options	MAPPIN-O_nurse3b_	MAPPIN-O_nurse3b_	MAPPIN-O_dyad3b_
	c) EBPI	To consider the criteria of evidence-based patient information	MAPPIN-O_nurse3c_	MAPPIN-O_patient3c_	MAPPIN-O_dyad3c_
Expectations and worries		To explore / discuss the patient’s expectations (*ideas*) and concerns (*fears*) about how to manage the problem	MAPPIN-O_nurse4_	MAPPIN-O_patient4_	MAPPIN-O_dyad4_
Indicate decision		To open the decision stage leading to the selection of an option	MAPPIN-O_nurse5_	MAPPIN-O_patient5_	MAPPIN-O_dyad5_
Follow up arrangements		To discuss plans for how to proceed	MAPPIN-O_nurse6_	MAPPIN-O_patient6_	MAPPIN-O_dyad6_
Preferred communication approach		To come to an agreement on the preferred mode of information exchange	MAPPIN-O_nurse7_	MAPPIN-O_patient7_	MAPPIN-O_dyad7_
Evaluation of understanding	patient	To clarify whether the patient correctly understood the information given by the nurse (clinician)	MAPPIN-O_nurse8_	MAPPIN-O_patient8_	MAPPIN-O_dyad8_
	nurse	To clarify whether the nurse (clinician) has correctly understood the patient’s point of view	MAPPIN-O_nurse9_	MAPPIN-O_patient9_	MAPPIN-O_dyad9_
Mean score of all indicators			MAPPIN-O_nursetotal_	MAPPIN-O_patienttotal_	MAPPIN-O_dyadtotal_

Observational data and the written feedback forms were analysed descriptively by two researchers (BBH and KL).

### Results

#### Sample

Two breast care centres agreed to participate (number of primary cases of DCIS in 2013: *N* = 40 and *N* = 19, respectively). Each centre had one BCN and one oncology nurse. The nurses were aged between 38 and 56 years and had an average of 15 to 35 years of nursing experience. Only the BCNs were fully excused from their work on the ward. The BCNs regularly participated in the tumour board meetings, although their function was mostly restricted to listening.

Five physicians participated. They were aged between 32 and 58 years and had between 9 and 30 years of work experience in the treatment of breast cancer.

Seven women with DCIS aged between 46 and 76 participated. All lesions were unifocal. Three women had high grade (grade III) and three intermediate grade DCIS; for one woman the grade was unknown.

#### Professionals’ expectations and worries before the intervention

Only nurses who were excused from their work on the ward considered that they would have enough time for coaching. The physicians appreciated the low threshold of decision-coaching by nurses. Only a few physicians were concerned that the information provided by nurses might be confusing for the patients. Splitting the decision-making process between professions was expected to be a potential time saver for physicians. The physicians rated patient involvement as important. They considered the tumour board recommendations as being in principle the best options for the patient, and ascribed high importance to it; however, they also stated that they could accept women choosing another treatment option.

#### Recruitment strategy for women with ductal carcinoma in situ

Within the study period, ten women with DCIS were eligible in both centres. The physicians were concerned that some of the women (*n* = 2) might be overburdened with information and hence did not include them. One woman declined because she had already made a final decision.

#### Women’s expectations and acceptance of decision-coaching

The women expressed a high level of social pressure to undergo treatment and feared they would die if they did not accept the recommended treatment prior to the intervention. They reported trust in the nurses’ competence before the decision-coaching. Due to the incorrect administration of the women’s structured feedback forms after decision-coaching, only three out of seven questionnaires were available for analysis, which was therefore omitted.

#### Women’s knowledge

Prior to decision-coaching, women’s estimations of their knowledge about breast cancer were heterogeneous. On average, the women had good knowledge of the risks of DCIS (score range 10–15 out of 15) after decision-coaching.

#### Patient involvement in treatment decision-making during decision-coaching

Seven decision-coaching sessions led by nurses were videotaped and analysed in terms of feasibility. On average, a basic level of ISDM was observed for nurses and patient–nurse dyads (M(MAPPIN-O_dyad_): 2.15 and M(MAPPIN-O_nurse_): 1.90 (see Table 3). Higher standards were observed for explaining and discussing the pros and cons of the different options and for considering the criteria for EBPI (M_indicator3b_(MAPPIN-O_dyad_): 2.86 and M_indicator3c_(MAPPIN-O_dyad_): 3.0).

**Table 3:** MAPPIN’SDM observer-based results (*N* = 7 decision-coaching sessions)

Indicator			Mean MAPPIN-O_nurse1-9_^a^ (Range)	Mean MAPPIN-O_patient1-9_^a^ (Range)	Mean MAPPIN-O_dyad1-9_^a^ (Range)
1	Defining problem		1.86 (1–3)	0.71 (0–1)	2.00 (1–3)
2	SDM key message		1.00 (1–1)	0.14 (0–1)	1.00 (1–1)
3	Discussing the options	a) structure	1.14 (0–3)	0.00 (0–0)	1.14 (0–3)
		b) content	2.71 (1–4)	2.29 (1–4)	2.86 (2–4)
		c) EBPI	3.00 (3–3)	1.43 (0–2)	3.00 (3–3)
4	Expectations and worries		2.29 (0–3)	3.00 (3–3)	3.00 (3–3)
5	Indicate decision		1.33 (0–2)	1.83 (0–3)	1.83 (0–3)
6	Follow up arrangements		1.83 (0–4)	1.33 (0–4)	1.83 (0–4)
7	Preferred communication approach		1.29 (0–2)	1.14 (0–2)	1.43 (0–2)
8	Evaluation of understanding	patient	2.14 (1–3)	2.86 (2–4)	3.00 (2–4)
9		nurse	2.14 (1–3)	2.00 (1–3)	2.43 (2–3)
	Mean Score of all indicators^a^ (MAPPIN-O_total)_		1.90 (1.27–2.64)	1.65 (1.22–2.42)	2.15 (1.73–2.73)

^a^Meaning of the score: o = The behaviour is not observed; 1 = The behaviour is observed as a minimal attempt; 2 = The basic competency is observed; 3 = The behaviour is observed to a good standard; 4 = The behaviour is observed to an excellent standard

Lower standards were observed for stating that there is more than one way to manage DCIS (SDM-key message) (M_indicator2_(MAPPIN-O_dyad_): 1.0), for opening the decision stage leading to the selection of an option (M_indicator5_(MAPPIN-O_dyad_):1.83) and discussing follow-up arrangements for treatment (M_indicator6_(MAPPIN-O_dyad_):1.83).

#### Use of the DA, patient guidance and fact sheets

The materials were used as intended during decision-coaching. The nurses judged the prompt cards and decision guidance as useful and supportive for decision-coaching. The physicians appreciated the DA, the decision guidance and the fact sheets.

#### Applicability to the procedures in breast care centres

The nurses estimated the duration of the first patient contact (delivering the DA) to be about 15 min. The time between the first nurse contact and the decision-coaching was between 2 and 7 days. The mean duration of the coaching consultation was 36 min (range 23–82 min). After the coaching, women were offered enough time to consider the treatment options. If a woman was already sure about her decision, the final physician consultation was arranged immediately after the decision-coaching. One physician appreciated the shorter consultations with patients after they had received the decision-coaching.

#### Professionals’ attitude toward the intervention

Two out of five physicians reported that the decision-coaching had improved patient involvement. One physician thought that the information process was neither simplified nor complicated by decision-coaching. The majority was convinced that women were better informed about treatment options compared to usual care. Some physicians perceived a greater reluctance in women to undergo radiation than previously. Nurses had the overall impression that women with DCIS benefitted from greater participation in treatment decision-making. The physicians and nurses reported successful inter-professional collaboration over the whole course of the study. The physicians reported increased trust in nurses’ competencies in decision-coaching. Nevertheless, one nurse felt overburdened by counselling women with DCIS. Most of the nurses appreciated the support of their colleagues and physicians.

#### Barriers and facilitators of the implementation

One nurse reported that ISDM was often hampered because the screening centres had already recommended specific treatments before the initial visit to the breast care centre, and the women felt obliged to follow these recommendations. The physicians emphasised the need for a clear-cut medical recommendation because they felt that was what patients wanted.

The nurses indicated that the main decisional conflict for women persisted in the decision between breast-conserving surgery with or without radiation. In all but one case, the physicians accepted the women’s decisions. Initially, one woman chose breast-conserving therapy without radiation as her preferred treatment option. Because this treatment option is not in line with medical guidelines, the woman was sent to a radiotherapist to discuss the treatment option with an expert. Finally, two women opted for watchful waiting, two for breast-conserving surgery without radiation, two for breast-conserving surgery with radiation and one for a mastectomy.

## Discussion

We developed and pilot tested a decision-coaching programme involving specialised nurses to implement ISDM in oncology. The pilot study showed that both women with DCIS and their physicians trusted the nurses’ counselling competencies. The women achieved good knowledge about DCIS and its treatment options, which is a prerequisite for making an informed choice [3].

The nurses attained basic levels of carrying out ISDM. Because the decision-making process was split between nurses and physicians, the nurses could not achieve maximum scores on some indicators of the MAPPIN’SDM instrument and thus lower scores do not indicate insufficient competencies. However, in the subsequent phase III study (cluster RCT) the training will need to focus more on certain indicators, such as the key message of SDM (see Table 2), although the mutual ISDM-behaviour of patients and nurses was already comparable with the physicians’ results in the IT’S SDM study, in which an RCT was conducted using the MAPPIN’SDM [49] to evaluate physicians’ SDM training.

We identified relevant barriers, such as healthcare professionals’ beliefs about the treatment options for DCIS, which are still the subject of controversial discussion among experts [50, 51]. ISDM may be hampered by the strong belief that treatment options other than those recommended by the tumour board and clinical guidelines put women at risk regarding prognosis. Shepherd et al. described similar concerns in a qualitative study with 22 physicians in different oncological settings [52]. In fact, uncertainty about the balance between the benefits and harms of treatment options should facilitate patient participation in decision-making. Therefore, we discussed these conflicts during the workshop, and explained that the various options are not equal in their risk-benefit profiles and women may use different decision-making criteria than those assumed by physicians; e.g., treatment efficacy and the need for safety versus overtreatment, breast conservation, short treatment duration or fewer adverse events. Assuming that women understand the information, taking their preferences into consideration may lead to informed decisions that differ from those recommended by physicians. Despite intensive reflection on their concerns, a few physicians were trying to persuade women whose treatment preferences differed from their own recommendations to follow the recommendation of the physician. However, finally we have seen that women chose various treatment options of which some differed from guideline recommendations. That might indicate that some women abided by their decisions.

We also identified the tumour board recommendation as a relevant structural barrier because it does not account for women’s individual preferences. Hahlweg et al. recently studied this barrier in different cancer centres at a German university hospital [45].

The physicians tended to select women according to their suitability for ISDM (e.g., age, education, diagnostic parameters). This is in line with other systematic reviews that identified relevant barriers to ISDM [53, 54], especially in oncology settings [55].

Although we revised the curricula according to the identified barriers, some barriers can only be solved by structural changes at the macro level; e.g., quality indicators that depict the number of informed treatment decisions or the amount of ISDM urgently needed [56], rather than quality indicators that define the proportion choosing a particular treatment procedure. In addition, misleading incentives emerge if a breast care centre’s profit depends on women’s decisions and if elaborated decision-coaching is not refunded. However, the financial costs of decision-coaching might be minimal, given that some physicians reported timesaving and that employing nurses for ISDM is cost-saving. This is an important advantage of decision-coaching because lack of time is a frequently reported barrier for the implementation of ISDM [55, 57].

Decision-coaching led by different healthcare professionals (e.g., pharmacists, nurses and psychologists) for different indications (e.g., genetic counselling, menopausal women) has been evaluated in several RCTs [58]. The results indicate increased knowledge and cost savings due to better health outcomes and less invasive and lower cost treatments than usual care. However, the effects on patient participation and decision-related outcomes, such as decisional conflict, remain unclear due to the heterogeneity of the results from such studies [58–60]. Moreover, decision-coaching in oncology settings, except for genetic counselling, has rarely been evaluated. To the best of our knowledge, our programme is the only inter-professional complex intervention in German oncology settings to involve nurses as decision coaches to implement ISDM. Evaluation of the efficacy of decision-coaching led by research psychologists in a German breast care centre showed no difference in decisional conflict compared with usual care [61]. Currently, a similar project is evaluating nurse-led immunotherapy decision-coaching in German outpatient clinics for multiple sclerosis [62].

A further strength of our study is the systematic, theory-based development and extensive piloting of the three components and then the entire intervention, involving relevant target groups. For example, we based our decisions regarding the content of the decision aid on the criteria for the development of evidence-based patient information (EBPI) [4] which includes the consideration of patient needs and is intended to enhance informed choices, whereby lacking evidence and concomitant uncertainties challenged the development process. Evidence-based DAs are a fundamental prerequisite for informed decision making, yet we identified a lack of evidence-based DAs during the development of our intervention. Although high-quality guidelines were available, they did not provide the necessary data for the DAs. In future studies, the synthesis of evidence during the guideline development processes should consider patients’ information needs and their current data that can be applied to the development of evidenced-based decision aids, as previously outlined by Mühlhauser et al. [63] and recommended by the European project DECIDE [64].

Despite its strengths, our study had several limitations. The breast care centres and the professionals were highly motivated to participate and the physicians may have been biased in selecting patients according to their characteristics. The study sample was very small and the results cannot be generalised. In particular, we failed to include women with DCIS in the early beginning of the development of our DA. Unfortunately, we had to refrain from the analysis of the women’s structured feedback forms after decision-coaching due to the incorrect administration in the participating centres. In addition, most of the women were higher educated. Therefore, we cannot be sure whether women feel satisfied with the intervention and whether lower educated women are adequately addressed. However, the small sample was appropriate for the study design and the results guided the formulation of the hypothesis that we are currently testing in a cluster RCT [65]. The research team critically discussed all of the results, but a systematic analysis of the data by two independent researchers was not feasible due to limited resources.

## Conclusions

Decision-coaching by specialised nurses in oncology is feasible in terms of the professional’s acceptance of the intervention, the applicability to the procedures in the breast care centres and the amount of patient involvement in treatment decision-making. Further research is needed to address the identified barriers and to clarify questions of generalizability to different subgroups e.g. women with low grade DCIS and women with lower health literacy and the perceived involvement in treatment decision-making by women, nurses and physicians. The complex intervention is currently being evaluated in a cluster randomised trial with 16 breast care centres (Current Controlled Trials ISRCTN46305518, date of registration: 05.06.2015) [65].

## Additional files

Additional file 1: Checklist of Criteria for Reporting the Development and Evaluation of Complex Interventions in healthcare (CReDECI 2). (DOCX 17 kb)

Additional file 2: Development process of the decision aid. (DOCX 45 kb)

Additional file 3: Learning objectives, content and educational strategies of the nursing curriculum. (DOCX 25 kb)

Additional file 4: Learning objectives, content and educational strategies of the physician workshop. (DOCX 18 kb)

## Abbreviations

AS: Anke Steckelberg
BBH: Birte Berger-Höger
BCN: Breast care nurse
DA: Decision aid
DCIS: Ductal carcinoma in situ
EBM: Evidence-based medicine
EBPI: Evidence-based patient information
IM: Ingrid Mühlhauser
IPDASi: International patient decision aids-instrument
ISDM: Informed shared decision making
KL: Katrin Liethmann
MAPPIN’SDM: Multifocal approach to ‘sharing’ in SDM
RCT: Randomised controlled trial
SPUPEO: Specialised nurses to support informed shared decision making in oncology (refers to the German translation of *Spezialisierte Pflegefachkräfte zur Unterstützung einer informierten partizipativen Entscheidungsfindung in der Onkologie*)
