# Supplementary material for: Implementation of shared decision-making in oncology: development and pilot study of a nurse-led decision-coaching programme for women with ductal carcinoma in situ
Source: BMC Med Inform Decis Mak. 2017 Dec 6;17:160. doi: 10.1186/s12911-017-0548-8 (PMC5719557; doi:10.1186/s12911-017-0548-8)
Supplement: Supplementary file 1 — Checklist of Criteria for Reporting the Development and Evaluation of Complex Interventions in healthcare (CReDECI 2). (DOCX 17 kb) [file 12911_2017_548_MOESM1_ESM.docx]

**Additional file 1: Checklist of Criteria for Reporting the Development and Evaluation of Complex Interventions in healthcare: revised guideline (CReDECI 2)**

Möhler et al. Trials (2015) 16:204 DOI 10.1186/s13063-015-0709-y

| **Stage** | **Reported on page** |
| --- | --- |
| **First stage: Development** | |
| 1. Description of the intervention’s underlying theoretical basis | Section: Methods, p. 5-6  Section: Development of the DA, p. 6-7  Section: Development of the curriculum for the nurse training, p.8-9  Section: Development of the physician workshop, p.13 |
| 1. Description of the single components of the intervention, including selection criteria as well as their aims / essential functions | Additional file 1-3  Section: Development of the DA, p.6-7  Section: Development of the curriculum for the nurse training, p.8-9  Section: Development of the physician workshop, p.13 |
| 1. Illustration of any intended interaction between components | Section: Methods, p.5-6 |
| 1. Description and consideration of the context’s characteristics in intervention modelling | Section: Pilot testing of the physician workshop, p.13-15 |
| **Second stage: Feasibility and piloting** | |
| 1. Description of the pilot test and its impact on the final version of the intervention | Section: Phase II Pilot testing of single components: DA p.7-8  Section: Pilot testing of single components and of the entire nurse training, p.9-13  Section: Pilot testing of the physician workshop, p.13-15  Section: Testing the entire intervention with women with DCIS, p.15-22 |
