# Supplementary material for: Implementation of shared decision-making in oncology: development and pilot study of a nurse-led decision-coaching programme for women with ductal carcinoma in situ
Source: BMC Med Inform Decis Mak. 2017 Dec 6;17:160. doi: 10.1186/s12911-017-0548-8 (PMC5719557; doi:10.1186/s12911-017-0548-8)
Supplement: Supplementary file 2 — Development process of the decision aid. (DOCX 45 kb) [file 12911_2017_548_MOESM2_ESM.docx]

**Additional file 2: Development process of the decision aid**

**[I] Search strategy for decision aids for women with breast cancer**

We searched in Medline, EMBASE, CINAHL, Psychinfo and the Cochrane Library for systematic reviews, randomized controlled trials (RCT) and controlled trials reporting the evaluation of DA in German and English. Publications were included until 13.06.2012.

Exclusion criteria comprised decision aids addressing genetic breast cancer treatment and prevention and treatment of advanced breast cancer. In addition, we searched for grey literature and on relevant homepages [1, 2].

Search Terms

choice behavior [Mesh]; decision making [Mesh]; decision support techniques[Mesh]; educational technology [Mesh]; decision*; choic*; preference*; communication package; health education [Mesh]; health knowledge, attitudes, practice; informed consent; patient participat*; consumer participat*; parent participat*; decision aid; shared decision making, informed choice, breast cancer, breast neoplasm*, clinical trial, randomized controlled trial, double blind

Two systematic reviews were identified [3, 4] and updated focussing on DA for breast cancer. 29 DA were identified. If DA were not accessible, authors were contacted (N=24). Two researchers critically appraised 27 DA using the International Patient Decision Aids (IPDAS)-instrument [5] and the EBPI-criteria [6].

7 out of 27 DA had been evaluated in RCTs.

**[II] Search strategy to identify the evidence base for the treatment of DCIS**

We conducted a systematic literature search in Medline, EMBASE and the Cochrane Library in order to identify the evidence base for the treatment of DCIS. The German guideline recommends breast conserving surgery with radiation or mastectomy for all women with DCIS [7]. Due to the risk of overdiagnosis and overtreatment, we added the options breast conserving surgery without radiation and watchful waiting. We included the best available evidence according to the principles of evidence-based medicine [8].

We included the following patient relevant outcomes: overall survival, ipsilateral recurrence of DCIS / invasive breast cancer, disease free survival, disease related quality of life. Adverse effects include overall rate of adverse effects, severe adverse events (common terminology criteria of adverse events grade 3 or 4) or frequent mild or moderate adverse events (CTCAE grade 1 or 2) and patient withdrawal due to adverse events. We excluded studies that studied mixed groups of breast cancer patients, lobular carcinoma in situ (LCIS), BRCA 1/2-mutation or men.

We also included studies that addressed secondary prevention of breast cancer/DCIS by lifestyle changes (e.g. smoking cessation) and predictors for the development of invasive cancer. The search was limited to English and German language. We included published studies up to 18^th^ July 2014. In addition, we reviewed medical guidelines.

**[III] Description of the DA**

For better readability and taking gender aspects into account, the text of the brochure has been written in the female form. Written information was illustrated by anatomical drawings and tables that summarise risks and benefits of treatment options. For treatment options which were evaluated in RCTs, the absolute risk reduction was displayed using pictograms. We explicitly differentiated in reporting results derived from RCTs and cohort studies. We stressed limitations of evidence due to lack of studies. For the design features of the value clarification tool see section IV. Women had access to an online series of pictures illustrating women who underwent surgery and breast reconstruction (http://www.spupeo.de/operative-verfahren/).

**Box 1: Main content of the DA**

**IV Structure of the decision guidance /value clarification tool**

| **Structure of the decision guidance** |
| --- |
| **A. Introduction**  Information about SDM and instructions for the decision guidance  **B Value clarification tool**   1. Inter-active part to document information of woman´s individual diagnostic parameters (e.g. size, grading, position of the DCIS) 2. Exploring women´s decision relevant criteria (e.g. breast conservation, to avoid long-term harms) 3. Treatment options with an interactive part to document women´s individual pros, cons and questions about each treatment option 4. Expectations, wishes and concerns 5. Decision making: Inter-active part of women´s requirements toward decision making and documentation of the preferred decision 6. Documentation of the decision implementation with the health care team 7. Reflexion of the decision and decision making process |
| **Type of value clarification method** |
| Splitted into a table with **pros and cons** which is the basis for **discussing** with the decision coach or physician and **thresholds** considering tradeoffs according to individual **outcomes or**  **processes**, e.g.: “For me it is important to avoid side effects” “The DCIS scares me” |
| **Position in decision aid -** Where is the Value clarification method placed in the decision aid? |
| Included in the DA after the information + additional component (decision guidance) |
| **Solo activity-** Is the value clarification method designed to be completed independently or with others? |
| Women complete the value clarification section within the decision coaching session; partly it can be completed independently |
| **Media -Which medium was used for the value clarification method?** |
| Paper – brochure |
| **Tradeoffs-** How are trade offs of preference-sensitive decisions presented? |
| Implied – table of pros and cons |
| **Visual metaphors -**Does the value clarification method use a visual metaphor as part of the design? |
| Strength of response – expectations, wishes and concerns – Likert Scale |
| **Open- or closed ended-** Were the sets of attributes presented to users closed-ended, open-ended or mixed? |
| Open-ended - the values clarification method is entirely open-ended, women generate their own list of attributes |
| **Elicitation Process -**What process the user might go through of the values clarification method to give responses to her values relevant to the decision? |
| Women are asked to list items that are relevant to the decision and matter to them |
| **Response Measure -**Which type of data is obtained via the elicitation process? |
| Non-numeric |
| **Values Exploration -**Does the value clarification method support an iterative discovery process of preferences to stabilize and if, how? |
| Revision is possible but not explicitly supported or encouraged |
| **Implications -**Does the value clarification method show users the implications of their expressed values? |
| Implications are not presented |
| **Decision Intentions -**Is a step included in which the user is asked to indicate her decision intention? |
| Yes (decision) |

**Box 1: Design features of the value clarification tool according to the taxonomy by Witteman et al. 2016 [9]**

**References**

1. Ottawa Hospital Research Institute: Patient Decision Aids Library. https://decisionaid.ohri.ca/ (2017) Accessed 27 Mar 2017.
2. Informed Medical Decisions Foundation**:** https://www.informedmedicaldecisions.org/ (2017). Accessed 27 Mar 2017.
3. Stacey D, Bennett CL, Barry MJ, Col NF, Eden KB, Holmes-Rovner M et al. Decision aids for people facing health treatment or screening decisions. Cochrane Database Syst Rev. 2012;CD001431.
4. Lenz M, Buhse S, Kasper J, Kupfer R, Richter T, Mühlhauser I. Decision aids for patients. Dtsch Arztebl Int. 2012;109(22-23):401-8.
5. Elwyn G, O'Connor AM, Bennett C, Newcombe RG, Politi M, Durand MA, et al. Assessing the quality of decision support technologies using the International Patient Decision Aid Standards instrument (IPDASi). *PLoS One.* 2009;4:e4705.
6. Bunge M, Mühlhauser I, Steckelberg A. What constitutes evidence-based patient information? Overview of discussed criteria. Patient Educ Couns. 2010;78(3):316-28.
7. Kreienberg R, Albert U-S, Follmann M, Kopp I, Kühn T, Wöckel A, et al. [Interdisciplinary S3-guideline on diagnostics, therapy and follow up of breast cancer]. In: German guideline program in oncology. 3rd ed. Berlin: AWMF, DKG, Deutsche Krebshilfe; 2012. http://leitlinienprogramm-onkologie.de/uploads/tx_sbdownloader/S3-Brustkrebs-v2012-OL-Langversion.pdf. Accessed 24 Mar 2017.
8. Sackett DL, Rosenberg WM, Gray JA, Haynes RB, Richardson WS. Evidence based medicine: what it is and what it isn't. BMJ. 1996;312(7023):71-72.
9. Witteman HO, Scherer LD, Gavaruzzi T, Pieterse AH, Fuhrel-Forbis A, Chipenda Dansokho S, et al. Design Features of Explicit Values Clarification Methods: A Systematic Review. Med Decis Making. 2016;36(4):453-471.
