# Supplementary material for: Implementation of shared decision-making in oncology: development and pilot study of a nurse-led decision-coaching programme for women with ductal carcinoma in situ
Source: BMC Med Inform Decis Mak. 2017 Dec 6;17:160. doi: 10.1186/s12911-017-0548-8 (PMC5719557; doi:10.1186/s12911-017-0548-8)
Supplement: Supplementary file 3 — Learning objectives, content and educational strategies of the nursing curriculum. (DOCX 25 kb) [file 12911_2017_548_MOESM3_ESM.docx]

**Additional file 3: Learning objectives, content and educational strategies of the nursing curriculum**

| **Module 1: Basics of medical decision making and judging the validity of information; 2 days, 14h + practical exercises 16h** | | |
| --- | --- | --- |
| **Objectives** | **Content** | **Educational strategies** |
| **Cognitive** | | |
| - Nurses describe and compare the different patient participation models paternalism, SDM and the autonomous decision model for medical decision making | - Models of medical decision making and patient participation: Paternalism, SDM and autonomous decision model | Lecture (presentation) and classification of a patient narrative |
| - Nurses explain the difference between the validity of individual case experience, expert knowledge and evidence-based knowledge using a report (film) about cryotherapy to avoid chemotherapy-induced alopecia. | - Individual case experience, expert knowledge, evidence-based knowledge - EBM | Problem based learning [1] using the example cryotherapy to avoid chemotherapy-induced alopecia: Observation task |
| - Nurses develop own ideas to evaluate the efficacy of cryotherapy considering key questions. |  | Group work: Design of a trial to evaluate the efficacy of cryotherapy |
| - Nurses describe EBM as a method. |  | Lecture (presentation) |
| - Nurses conclude that a control group is needed to verify efficacy of cryotherapy by comparing their preliminary considerations and two different trial summaries. | - Efficacy verification by RCT - Control vs. intervention group | Group work: Trial summaries and discussion of the results. |
| - Nurses identify the necessity of randomised controlled trials to show efficacy of the intervention. |  |  |
| - Nurses explain quality criteria of randomized controlled trials. | - Blinding - Randomization - Selection criteria and bias (Intention to treat analysis) - Patient relevant outcomes | Work stations |
| - Nurses assess the quality of a randomized controlled trial comparing breast conserving surgery vs. breast conserving surgery and radiation for the treatment of DCIS. |  | Group work: Problem based embedded in a patient story: Application to the example breast conserving surgery vs. breast conserving surgery and radiation for the treatment of DCIS |
| - Nurses derive information from the trial results for the communication of effectiveness of adjuvant radiation with patients. |  |  |
| - Nurses describe evidence-based medical guidelines as an implementation tool of evidence-based knowledge for practitioners using the example of the German breast cancer guideline (S3). | - Guidelines | Lecture (presentation) |
| - Nurses explain the difference between the absolute and the relative risks of benefits and harms using the example of breast conserving surgery and radiation for the treatment of DCIS. | - Relative risk - Absolute risk - Relative risk reduction | Lecture |
| - Nurses define their own criteria for high quality patient information. | - Criteria of evidence-based patient information - Critical appraisal of EBPI | Experience based (Scheller) [2]  Group work (Appropriation of experiences) |
| - Nurses describe their previous search strategies to identify patient information material about therapies and adverse effects. |  |  |
| - Nurses elucidate the criteria of evidence-based patient information. |  | Lecture and critical appraisal of patient information material |
| - Nurses appraise commonly used patient information material like patient guidelines or brochures of the German cancer aid with the criteria of EBPI and their appropriateness to support informed decision making. |  |  |
| - Nurses explain the difference of EBPI and medical decision aids. | - Decision aids | Lecture |
| **Psychomotor** | | |
| - Nurses calculate the absolute and relative risks of harms and benefits which are reported in the SPUPEO decision aid and communicate risks in plain language. | - Relatives risk - Absolute risk - Absolute and relative risk reduction | Exercise: Calculation of risks using the example breast conserving surgery and radiation |
| **Affective** | | |
| - Nurses critically reflect the model of informed choice with regard to their personal and work experience. | - Strategies of decision making | Experience based[2]: Reflection of a personal decision (Single exercise in written form) |
| - Nurses are aware of insufficient patient information and ways to enhance patient participation in medical decision making considering an example of a women with breast cancer | - Opportunities of patient participation in medical decision making - Decisional conflict | Confrontation with an original patient experience (narrative) and discussion |
| - Nurses reflect the information-sharing behaviour with patients in their daily practice and potentially associated problems derived from the example of cryotherapy. | - Models of medical decision making and patient participation - Individual case experience, expert knowledge, evidence-based knowledge | Group work: Trial summaries and conclusions |
| - Nurses recognize the additional value of EBPI by contrasting conventional patient information with EBPI | - EBPI - Evidence bases decision aids | Homework: Critical appraisal of their previously used information material and the decision aid of DCIS |
| ***Knowledge questionnaires*** | | |
| **Module 2: SDM and decision coaching; (1 day 7h, according to the group size up to two days)** | | |
| **Objectives** | **Content** | **Educational strategies** |
| ***Knowledge questionnaires*** | | |
| **Cognitive** | | |
| - Nurses elucidate the distinct steps of SDM using the SPUPEO prompt cards. | - Steps of SDM | Lecture and private study |
| **Psychomotor** | | |
| - Nurses explain the content of the DA in a correct and understandable manner for women with DCIS. | - DA DCIS | Lecture  Role play |
| - Nurses take part in tumour boards and patient physician consultations and emphasize their role in the inter-professional team. | - Nurse´s role in the tumour board and physician consultation | Homework: Attendance of the tumour board and patient physician consultation  Discussion |
| - Nurses conduct a decision coaching using prompt cards, the decision guidance, the information sheets and the decision aid for DCIS and give feedback to each other. | - SDM - Decision guidance - Information sheets | Role play and feedback |
| **Affective** | | |
| - Nurses reflect their previous role in the multi-professional team and medical treatment decision making. | - Nurse´s role in the tumour board and physician consultation | Homework: Attendance of the tumour board and patient physician consultation  Discussion |
| ***Role play with feedback*** | | |
| ***Knowledge questionnaires*** | | |
| **Outcome objectives** | | |
| Women with DCIS get informed about treatment options by the decision coach through decision guidance and decision aid. | | |
| Women with DCIS make informed decision based on evidence-based knowledge and according to their personal preferences. | | |
| Decision coaches are embedded as a stable part in the decision making process and work closed with other professions. | | |

**References**

1. Roth H. [Pedagogical psychology of teaching and learning]*.* 11th edn. Berlin, Darmstadt, Dortmund, Hannover: Schroedel; 1969.

2. Scheller I. [Expericence based edcuation – Practice, design, theory]*.* 2nd ed. Frankfurt am Main: Scriptor; 1987.
