# Supplementary material for: Implementation of shared decision-making in oncology: development and pilot study of a nurse-led decision-coaching programme for women with ductal carcinoma in situ
Source: BMC Med Inform Decis Mak. 2017 Dec 6;17:160. doi: 10.1186/s12911-017-0548-8 (PMC5719557; doi:10.1186/s12911-017-0548-8)
Supplement: Supplementary file 4 — Learning objectives, content and educational strategies of the physician workshop. (DOCX 18 kb) [file 12911_2017_548_MOESM4_ESM.docx]

**Additional file 4: Learning objectives, content and educational strategies of the physician workshop**

| **Physician Workshop** | | |
| --- | --- | --- |
| **Objectives** | **Content** | **Educational strategies** |
| - Physicians describe the SDM-concept, criteria of an informed choice and their role in the decision making process. | - Steps of SDM - Informed choice | Lecture and discussion |
| - Physicians describe the information of the decision aid considering an adequate risk communication for women with DCIS. | - DA DCIS - Relatives risk / Absolute risk - Absolute and relative risk reduction | Lecture and discussion |
